# Influence of Dietary Rumen-Protected Choline and Nicotinic Acid Supplementation on Growth Outcomes and Meat Tenderness, Rumen Microbial Composition, and Serum Metabolomics of Late-Fattening Beef Cattle

**DOI:** 10.3390/ani16193144

**Published:** 2026-10-08

**Authors:** Baochen Xu, Tianyu Gao, Xiaoyan Wang, Mengqi Li, Xuekuan Hui, Yanxia Xing, Yang Li, Lizeng Peng, He Zhu, Dong Chen

**Affiliations:** 1 College of Modern Beef Cattle Industry, College of Food Science and Engineering, Shandong Agriculture and Engineering University, Jinan 250100, China; z2025094@sdaeu.edu.cn (B.X.); 2026121131@sdau.edu.cn (T.G.); 20252217022@stu.qau.edu.cn (M.L.); z2017014@sdaeu.edu.cn (X.H.); z2014007@sdaeu.edu.cn (Y.X.); z2013501@sdaeu.edu.cn (Y.L.); 2 College of Food Science and Engineering, Shandong Agricultural University, Tai’an 271018, China; 3 College of Animal Science and Technology, Hunan Agricultural University, Changsha 410128, China; ssnyn26@163.com; 4 College of Food Science and Engineering, Qingdao Agricultural University, Qingdao 266109, China; 5 Key Laboratory of Novel Food Resources Processing, Institute of Agro-Food Sciences and Technology, Shandong Academy of Agricultural Sciences, Jinan 250100, China; penglizeng@sdnu.edu.cn

**Keywords:** beef cattle, rumen-protected choline, nicotinic acid, rumen microbiota, serum metabolomics

## Abstract

High-concentrate feeding during late fattening can impair metabolic health and meat quality in beef cattle, yet effective nutritional strategies remain limited. Choline and nicotinic acid are involved in lipid and energy metabolism, but information on their combined or sequential supplementation is scarce. This study evaluated rumen-protected choline given alone or together with nicotinic acid on growth performance, meat quality, serum biochemical indices, serum metabolomics, and free amino acid profiles in late-fattening beef cattle. Twenty-four black cattle were randomly allocated to three groups: CON (basal diet), CC (basal diet + 10 g/d RPC for 90 d), and CC-CNA (basal diet + 10 g/d RPC for 90 d + 10 g/d protected NA during the final 30 d). Shear force was significantly lower in both CC and CC-CNA than in CON (*p* < 0.05), indicating improved tenderness. Exploratory serum metabolomics identified treatment-associated metabolites and pathways related to amino acid, purine, sphingolipid, and lipid metabolism. Taste-related free amino acids were also altered by supplementation (*p* < 0.05). RPC alone or with short-term NA may improve tenderness and modulate systemic metabolic responses; larger studies with direct sensory evaluation are needed to confirm growth and flavor-related effects.

## Simple Summary

High-concentrate feeding can impair health in finishing beef cattle and may limit production performance. This study therefore evaluated dietary rumen-protected choline alone or combined with nicotinic acid during late fattening, with emphasis on growth-related outcomes and serum biochemical indices. Supplementation with rumen-protected choline and nicotinic acid improved meat quality, enhanced antioxidant capacity and promoted overall health in cattle. These findings provide a basis for investigating the mechanisms by which choline alone and choline combined with nicotinic acid affect cattle health and support scientifically informed feeding practices in beef cattle production.

## 1. Introduction

Improving growth performance, meat quality, and metabolic health is a major goal of late-fattening beef cattle production. To meet production targets and consumer demand, high-concentrate diets are commonly used during fattening [1]. Feed utilization efficiency and host metabolism, however, are strongly influenced by the rumen microbiota, whose composition and function can be modified through dietary interventions, including supplementation with specific bioactive nutrients [2]. Improving the nutritional quality of ruminant diets may therefore enhance meat production performance and product quality [3]. Choline and nicotinic acid have established functions in lipid metabolism, methyl-group donation, neurotransmitter synthesis, redox balance, and energy homeostasis [4].

Rumen-protected choline (RPC) supports several physiological functions, including cell membrane formation, acetylcholine biosynthesis, lipid metabolism, and cell signaling [5]. Evidence from ruminants indicates that choline supplementation can influence meat-quality traits. Li et al. [6] found that RPC reduced shear force in lamb meat, raised the concentrations of umami, essential, and total amino acids in the meat, while lowering its unsaturated-fatty-acid fraction. In goats, Tu et al. [7] reported lower drip and cooking losses together with shear force after supplementation with 0.2% RPC. Li et al. [8] likewise observed higher lamb meat pH together with reduced drip loss and shear-force values after 0.25% RPC. Although the effective doses and responsive traits differed among these studies, the available evidence suggests that dietary choline may improve tenderness, water-holding capacity, and nutritional composition in ruminant meat.

Nicotinic acid (NA), also known as niacin, is a naturally occurring form of vitamin B3 [9]. It is the metabolic precursor of NAD/NADH and NADP/NADPH and is thus involved in biochemical pathways associated with lipid metabolism, oxidative tissue functions, glycolysis and respiration [10]. Dietary NA influenced lipid metabolism in cattle [11], and supplementation of 1000 mg/kg improved marbling score and increased intramuscular lipid levels in the longissimus dorsi muscle. Zhang et al. [9] reported that dietary supplementation of NA in geese increased intramuscular fat and reduced shear force, and regression analysis indicated that the optimum NA supplementation range was 80–90 mg/kg. In addition, Jiang et al. [12] reported that the inclusion of NA at the level of 60 mg/kg in poultry feed improved the daily weight gain and reduced the drip loss of breast muscle. Altogether, these studies indicate that NA might be a dietary supplement to enhance meat quality and productivity of meat-producing livestock.

Individual effects of choline and nicotinic acid in livestock nutrition have been documented [13,14], whereas information on their combined supplementation remains scarce. In particular, little is known about integrated responses involving meat quality, rumen microbial ecology, and systemic metabolism in cattle during late fattening. To address this gap, the present study assessed rumen-protected choline given alone or together with nicotinic acid and evaluated growth outcomes, meat-quality traits, rumen microbiota, serum metabolomics, and free amino acid profiles in late-fattening cattle for beef production.

## 2. Materials and Methods

### 2.1. Ethical Approval and Compliance

All animal experiments were performed according to the Chinese Guidelines for Animal Welfare and were approved by the Institutional Animal Care and Use Committee of Hunan Agricultural University (HNAU-LSK-23-263). The experimental procedures were carried out in accordance with the requirements from the approved Regulations for the Administration of Affairs Concerning Experimental Animals, by the State Science and Technology Commission of P. R. China (1988).

### 2.2. Study Design and Animal Husbandry

This dietary experiment was performed at the facilities of Shandong Qantas Wagyu Farming Company Limited (Zibo, China). A total of 24 healthy black cattle (castrated males), each 25 months old, with comparable body weights and a consistent genetic background (Wagyu × Luxi yellow cattle), were allocated at random among three treatment groups containing eight animals each. CON cattle received only the basal diet; CC cattle received the basal ration containing 10 g/d rumen-protected choline; and CC-CNA cattle received rumen-protected choline together with rumen-protected nicotinic acid according to the treatment schedule. The basic diet was formulated according to The Feeding Standard for Beef Cattle (NY/T 815-2004) [15] with concentrate and roughage at a ratio of 8:2 and the composition and nutrients of the ration are shown in Table 1. The quantities of choline and nicotinic acid were measured according to previous research [16,17]. CC-CNA subjects were fed rumen-protected choline from day 1 to day 60 of the 90 d feeding period and a combination of rumen-protected choline and rumen-protected nicotinic acid during the last 30 d of the study. The experiment lasted for a total of 100 days including a 10-day adaptation period. Rumen encapsulated choline (32% concentration; Hunan Pfik Biotechnology Co., Ltd.) (Changsha, China) and rumen encapsulated nicotinic acid (45% concentration; Vetagro S.p.A., Reggio Emilia, Italy) were used. Animals were housed individually in the same barn, and were fed daily at 07:00 and 16:00 with free access to drinking water. Additional research materials and reagents were provided by Shandong Qantas Wagyu Animal Husbandry Co., Ltd. (Zibo, China).

### 2.3. Beef Sample Collection

At the end of the experimental period, the cattle were fasted for 24 h and then slaughtered. Approximately 2000 g of Longissimus lumborum muscle was collected from each specimen. The meat color, shear force, cooking loss and carcass weight parameters were then evaluated and recorded. Additional muscle tissue was frozen rapidly in liquid nitrogen, and then transported to the laboratory for standard nutrient analysis. The whole rumen content of each animal was immediately collected and homogenized after slaughter. The liquid-associated microbial community was obtained through four layers of gauze. The filtrate was placed in 15 mL centrifuge tubes and then stored at −20 °C for DNA extraction and 16S rRNA sequencing. Blood was collected from the jugular vein at slaughter after a 24 h fast, allowed to sit undisturbed for 15 min, and then centrifuged at 4000 revolutions per minute for an additional 15 min, after which the resultant serum supernatant was placed into 1.5 mL tubes for storage at −20 °C until analysis.

### 2.4. Growth Performance and Meat Quality Determination

Body weights at trial initiation (IBW) and completion (FBW) were documented, and average daily gain (ADG) was calculated across the 90-day experimental period. Within 45 min after slaughter, Longissimus lumborum pH was determined using a calibrated Testo 205 pH meter (Testo SE & Co. KGaA, Titisee-Neustadt, Germany); three readings were obtained and subsequently averaged for each sample. Color was characterized using the L*, a*, and b* coordinates for lightness, redness, and yellowness, respectively, with a CR400 chromometer (Konica Minolta, Tokyo, Japan). Triplicate measurements were obtained at three randomly selected positions on each sample and averaged for analysis. Intramuscular fat content (ether extract, EE) was quantified following GB/T 6433-2006 [18] and AOAC methods [19]. For cooking-loss analysis, a 25–50 g muscle portion from each animal was trimmed of connective tissue and external fat, then immersed in an 85 °C water bath until the center temperature reached 70 °C. Samples were allowed to cool for 2 h, reweighed, and used to calculate cooking loss according to Equation (1):

(1) $$\mathrm{Cooking}\;\mathrm{loss}\;\left(\%\right)=\frac{\left(\mathrm{Pre}-\mathrm{cooking}\;\mathrm{weight}-\mathrm{post}-\mathrm{cooking}\;\mathrm{weight}\right)}{\mathrm{Pre}-\mathrm{cooking}\;\mathrm{weight}}\times 100\;$$

After cooking loss was determined, 1 cm × 1 cm × 1 cm muscle specimens were prepared parallel to the muscle-fiber orientation with a surgical blade. Shear force was determined perpendicular to the fibers with 1.0 mm/s test speed (CL-ML3 muscle tenderness meter). Three measurements were obtained for each sample and averaged.

### 2.5. Analysis of Serum Biochemical Parameters

Serum concentrations of non-esterified fatty acids (NEFA), triglycerides (TG), and blood urea nitrogen (BUN), together with alanine aminotransferase (ALT), superoxide dismutase (SOD), glutathione peroxidase (GSH-Px), and malondialdehyde (MDA), were assayed following the manufacturer’s protocol supplied by Beijing Andy Huatai Biotechnology Co., Ltd. (Beijing, China). Precision: intra-assay < 8% and inter-assay < 10%.

### 2.6. Metabolite Extraction

Serum was equilibrated at 4 °C following removal from −20 °C storage. A 100 µL aliquot of each sample was combined with 700 µL of extraction solvent comprising the internal standard mixture (methanol: acetonitrile: water, 4:2:1, *v*/*v*/*v*). After 1 min of vortex mixing, samples remained at −20 °C over a 2 h period before 15 min centrifugation at 25,000× *g* while maintained at 4 °C. Next, the 600 µL supernatant aliquot was placed inside an EP tube for drying. The dried extract was redissolved in 80 µL of a methanol/water mixture (1:1, *v*/*v*), mixed by vortexing for 10 min before being subjected to centrifugation once more at 25,000× *g* over a further 15 min. Supernatants from this final step were loaded into sample vials prior to UPLC-MS detection. A pooled quality-control (QC) sample was generated from 20 µL aliquots of all processed samples and analyzed throughout the sequence.

### 2.7. UPLC-MS Detection Conditions

Metabolite separation and detection were carried out on a Waters UPLC I-Class Plus interfaced with a Q Exactive high-resolution mass spectrometer from Thermo Fisher Scientific (Waltham, MA, USA). Chromatographic resolution employed a BEH C18 analytical column (1.7 μm, 2.1 × 100 mm; Waters, Milford, MA, USA). During positive-ion acquisition, phase A comprised 0.1% formic acid in water, whereas phase B consisted of methanol with formic acid at 0.1%. During negative-ion acquisition, phases A and B contained 10 mmol/L ammonium formate dissolved in water together with 10 mmol/L ammonium formate in 95% methanol, respectively.

Elution followed this gradient: 2% B from 0–1 min; a programmed increase from 2% to 98% B during 1–9 min; 98% B from 9–12 min; a return from 98% to 2% B over 12–12.1 min; and 2% B from 12.1–15 min. The system was operated at a flow rate of 0.35 mL/min with a column temperature of 45 °C and an injection volume of 5 μL. Mass spectra were recorded in the mass range *m*/*z* 70–1050. The resolutions of the primary and secondary scans were 70,000 (AGC 3e6; injection time, 100 ms) and 17,500 (AGC 1e5; injection time, 50 ms), respectively.

### 2.8. Metabolomics Data Processing and Quality Control

Raw metabolomic datasets were analyzed by Compound Discoverer version 3.3 (Thermo Fisher Scientific, USA). The analytical process consisted of peak detection and alignment, retention time correction, deconvolution, background signal subtraction, missing value imputation and peak area normalization.

Pooled QC samples were distributed across the run sequence to evaluate system stability. Analytical reproducibility was examined using PCA, overlap of total-ion chromatograms, and RSD of peak areas. Features exceeding 30% RSD were removed. The remaining normalized data underwent log transformation and Pareto scaling before downstream statistical evaluation.

Metabolite annotation relied on exact mass, retention time, isotopic pattern, and MS/MS spectra, with matches searched against HMDB, KEGG, LipidMaps, mzCloud, ChemSpider, together with an internal library at a mass tolerance < 5 ppm. Data from positive- and negative-ion acquisition were processed independently before being combined into a nonredundant table. For compounds detected in both modes, the retained feature was selected on the basis of superior MS/MS matching and peak shape together with a lower RSD. KEGG was used for subsequent pathway enrichment analysis.

### 2.9. 16S rRNA Gene Sequencing and Bioinformatic Analysis

Rumen-content samples were used for extraction of microbial genomic DNA. The V3-V4 hypervariable segment of the bacterial 16S rRNA gene was PCR-amplified using primer 338F (5′-ACTCCTACGGGAGGCAGCA-3′) together with 806R (5′-GGACTACHVGGGTWTCTAAT-3′). After purification, the resulting amplicons underwent 2 × 300-bp paired-end sequencing with an Illumina MiSeq platform (Illumina, San Diego, CA, USA).

Quality filtering. Raw reads were processed as follows: (1) a 25 bp sliding window was used, and bases were truncated from the 3′ end when the average quality score in the window fell below 20; reads shorter than 75% of the original length were discarded; (2) adapter-contaminated reads were removed (15 bp overlap, up to 3 mismatches allowed); (3) reads containing N bases were removed; and (4) low-complexity reads (continuous identical bases ≥ 10) were removed. Barcode and primer sequences were required to match with 0 mismatches.

Read merging. Paired-end reads were merged into tags using FLASH v1.2.11, with a minimum overlap of 15 bp and a maximum mismatch rate of 0.1 in the overlapping region.

OTU clustering and chimera removal. Tags were clustered into OTUs at 97% sequence similarity using USEARCH v7.0.1090 (UPARSE pipeline). Chimeras were removed from OTU representative sequences using UCHIME v4.2.40 against the gold database (v20110519). All tags were then mapped back to OTU representative sequences using the usearch_global method to generate the OTU abundance table.

Taxonomic assignment. The resulting OTUs were taxonomically assigned with RDP Classifier 2.14 against RDP training set No. 19, using 0.8 as the confidence cutoff.

### 2.10. Quantification of Free Amino Acids

Individual free amino acid concentrations were quantified following the procedure of Sun et al. [20]. In brief, a 5 g portion of beef was added to 5 mL of 10% TCA, homogenized for three 10 s intervals at 3000 rpm, before being subjected to centrifugation at 8000× *g* for 10 min. The resulting precipitate underwent five washes with 5% TCA before filtration through a 0.45 μm membrane filter. Prepared samples underwent analysis on an automated amino-acid analyzer.

### 2.11. Statistical Procedures

All data were processed for statistical evaluation in SPSS version 22.0. First, the normal distribution of data and homogeneity of variance were assessed according to residual plots, and data with abnormal distributions were normalized using log-transformations. Treatment effects were tested by one-factor ANOVA with Duncan’s multiple-range procedure for subsequent pairwise comparisons. Results are reported as means ± SEM. A threshold of *p* < 0.05 defined a significant effect, whereas *p* < 0.01 denoted a highly significant effect, and values from 0.05 ≤ *p* < 0.10 were treated as a tendency.

For metabolomics, between-group changes were evaluated by combining fold-change (FC) estimates with Student t testing. VIP values were obtained from the OPLSDA model. Differential status required fulfillment of all three thresholds: VIP ≥ 1.0, |log2FC| ≥ 1.0 (equivalent to FC ≥ 2 or ≤0.5), and *p* value of FDR correction < 0.05.

## 3. Results

### 3.1. Growth Performance

No statistically meaningful treatment effects were found for IBW, FBW, or ADG across the CON, CC, and CC-CNA groups (*p* > 0.05). Nonetheless, the numbers of CC and CC-CNA groups were higher than those of CON (Table 2), but this nonsignificant numerical difference warrants further investigation.

### 3.2. Serum Biochemical Indices

Dietary RPC and NA supplementation significantly affected serum biochemical indices in beef cattle (Table 3). Relative to CON, NEFA and BUN concentrations and ALT activity were significantly reduced in the CC and CC-CNA groups, whereas SOD and GSH-Px activities showed significant increases. TG and MDA did not differ significantly between CON and CC. The CC-CNA group had significantly higher TG than both CON and CC, while its MDA concentration was significantly lower than that of CON.

### 3.3. Serum Metabolomic Analysis

#### 3.3.1. Differential Metabolite Abundance and Comparative Analysis

**Figure 1:**
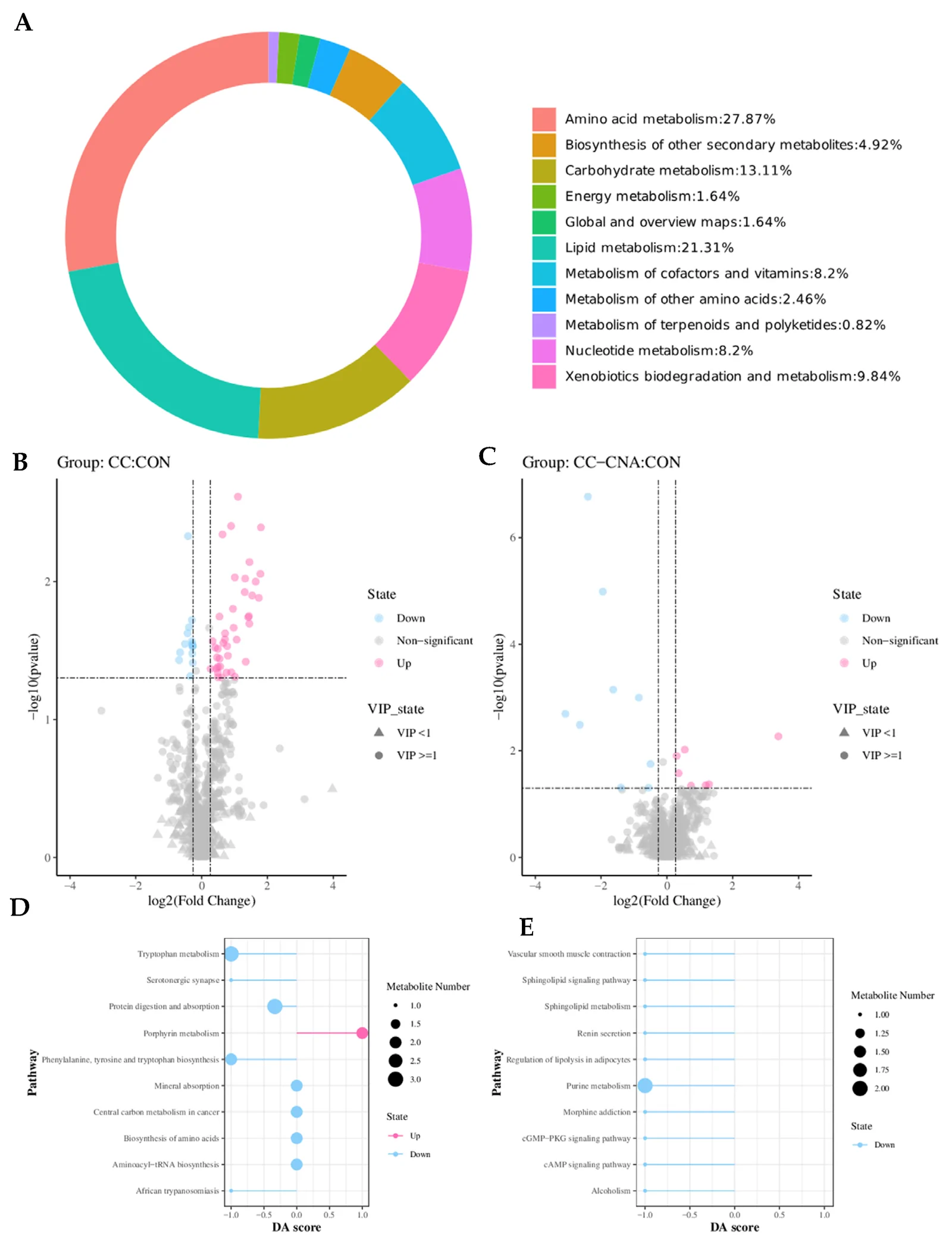
Serum-based analysis of differential metabolite abundance and metabolic pathways. (**A**) KEGG pathway classification of annotated serum metabolites; (**B**) volcano diagram comparing differential metabolites in CC versus CON; (**C**) volcano diagram comparing differential metabolites in CC-CNA versus CON; (**D**) enriched metabolic pathway differential-abundance scores comparing CC versus CON; (**E**) enriched metabolic pathway differential-abundance scores comparing CC-CNA versus CON.

Serum metabolomic profiling initially detected 2091 features under positive- and negative-ion acquisition modes. Signals showing relative standard deviation (RSD) above 30% in pooled quality-control (QC) samples were excluded before database matching. Following filtering, 791 metabolites remained with reliable annotations. KEGG classification placed these annotated metabolites into 11 metabolic categories (Figure 1A). Metabolism of amino acids (27.87%) together with lipid metabolism (21.31%) represented the two largest categories, followed by carbohydrate metabolism (13.11%), xenobiotics biodegradation and metabolism (9.84%), and nucleotide metabolism (8.20%).

Candidate metabolites were selected using VIP of at least 1.0, |log_2_(Fold Change)| of at least 1.0 (i.e., fold-change values ≥ 2 or ≤0.5), together with *p* < 0.05 by Student’s *t*-test. In these volcano plots (Figure 1B,C), green, red, and gray points represent significantly downregulated, significantly upregulated, and nonsignificant metabolites, respectively. Comparison of CC with CON identified 55 differential metabolites, comprising 40 upregulated and 15 downregulated compounds (Figure 1B). Comparison of CC-CNA with CON identified 16 differential metabolites, among which 7 were increased and 9 were decreased (Figure 1C). These compounds may serve as candidate biomarkers of metabolic responses to choline alone or in combination with nicotinic acid.

#### 3.3.2. KEGG Pathway Enrichment of Differential Metabolites

The identified differential metabolites were further analyzed for KEGG-based pathway enrichment (Table 4). For CC compared with CON, L-tryptophan, L-proline, and indole constituted the principal differential metabolites, with enrichment in pathways for protein digestion and absorption, tryptophan metabolism, aminoacyl-tRNA biosynthesis, mineral absorption, phenylalanine/tyrosine/tryptophan biosynthesis, amino acid biosynthesis, and serotonergic synapse. For CC-CNA compared with CON, the principal metabolites were adenosine, uric acid, and phytosphingosine. The corresponding enriched pathways included purine metabolism, cGMP-PKG signaling and regulation of adipocyte lipolysis, sphingolipid-related signaling and metabolism, vascular smooth muscle contraction, renin secretion, and AMPK signaling.

The enriched-pathway patterns for the CC vs. CON and CC-CNA vs. CON comparisons are presented in Figure 1D,E. As shown in Figure 1D, among pathways enriched for CC versus CON, metabolites annotated to porphyrin metabolism were the only upregulated set, whereas those annotated to the remaining pathways were downregulated. Figure 1E shows downregulation of metabolites assigned to the enriched pathways for CC-CNA versus CON. These results indicate distinct metabolic changes associated with choline supplementation alone and with combined choline–nicotinic acid supplementation.

Serum metabolomic analysis helped characterize physiological responses to the two supplementation regimens. Under the CC treatment, enrichment of protein-digestion and absorption, tryptophan metabolism together with aminoacyl-tRNA biosynthesis was consistent with possible changes in amino acid metabolism and nitrogen utilization. Under CC-CNA, enriched purine metabolism, cGMP-PKG signaling, regulation of adipocyte lipolysis, and sphingolipid metabolism indicated involvement of energy homeostasis, lipid metabolism, and cellular signaling. Adenosine, identified as a key differential metabolite, has established links with energy regulation and intercellular communication. Disease- and addiction-related names appearing in KEGG output should not be interpreted as evidence of disease in cattle because these terms originate from pathway database annotation.

### 3.4. Analysis of Rumen Microbial Diversity

#### 3.4.1. Richness and Diversity Estimators of Rumen Bacterial Communities

Sequencing coverage exceeded 99% in all experimental groups, as indicated by Good’s coverage (Table 5), supporting adequate depth for characterization of rumen bacterial richness and structural diversity. None of the Sobs, Chao1, ACE, Shannon, Simpson, or Good’s coverage indices differed significantly among treatments (*p* > 0.05). Accordingly, neither CC nor CC-CNA supplementation produced a significant change in rumen microbial alpha diversity.

#### 3.4.2. Relative Abundance (%) Within Rumen Bacterial Communities at Phylum and Genus Levels

**Figure 2:**
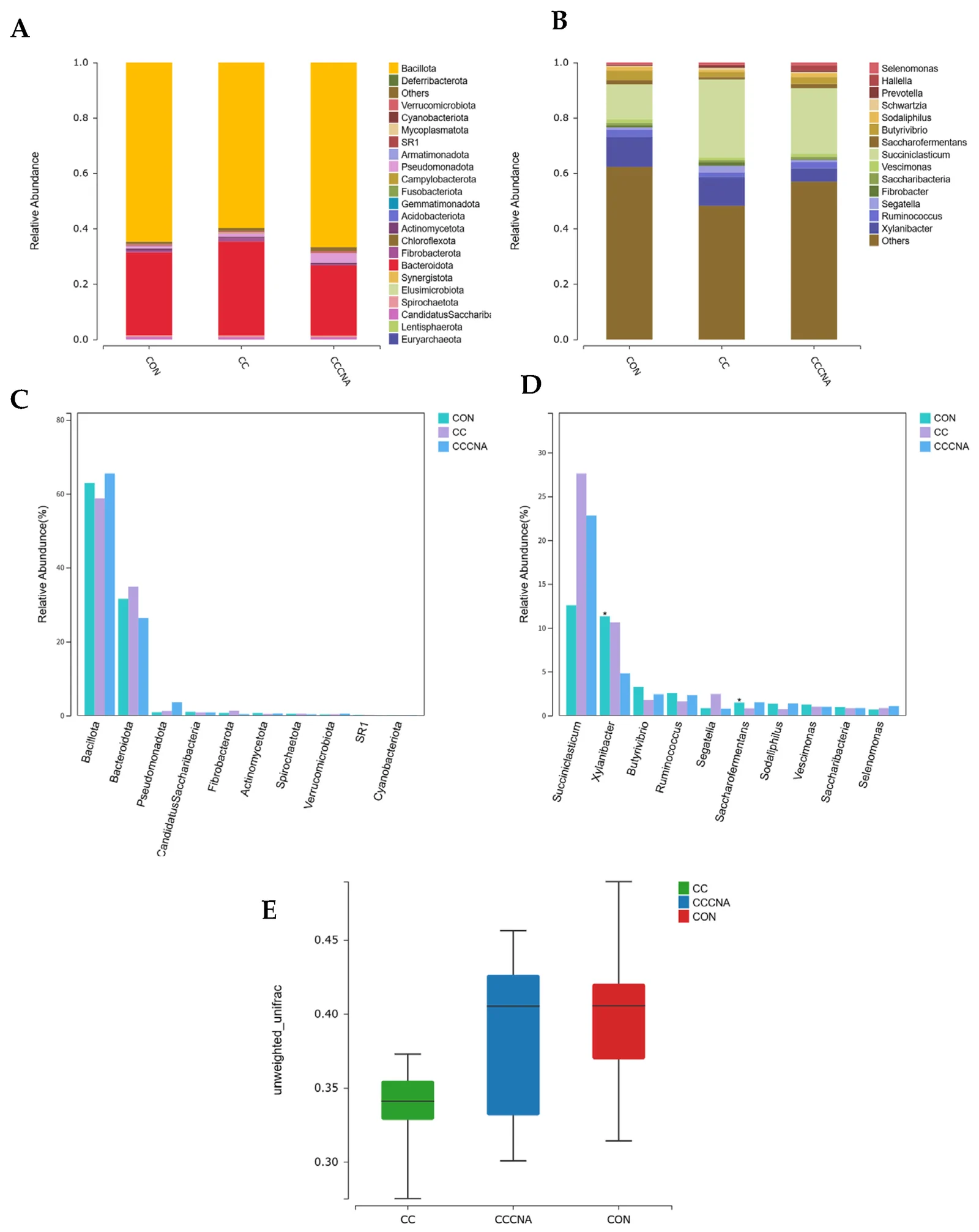
The rumen bacterial community: (**A**) phylum-level taxa; (**B**) genus-level taxa; (**C**) significance analysis at the phylum-level; (**D**) significance analysis at the genus-level; (**E**) beta-diversity analysis. * Representing significant differences within this group.

RDP-based classification at the phylum level identified 23 phyla across the three experimental groups (Figure 2A). Bacillota was predominant in every group and represented 58.77–65.51% of all sequences. Bacteroidota ranked second in relative abundance, accounting for 26.36–34.93%.

For the genus-level analysis (Figure 2B), comparison with the RDP database identified 15 bacterial genera across the three experimental groups. Succiniclasticum was the dominant genus in every group and represented 12.59–27.61% of total sequences. Xylanibacter ranked second, accounting for 4.19–10.62%. The 16S rRNA gene sequencing approach used in this study cannot exclude all potential biases inherent to amplicon-based methods. The relative abundances reported will be cautiously explained in the following text and validated in future studies using metagenomic or quantitative approaches.

### 3.5. Free Amino Acid Content in Meat

**Figure 3:**
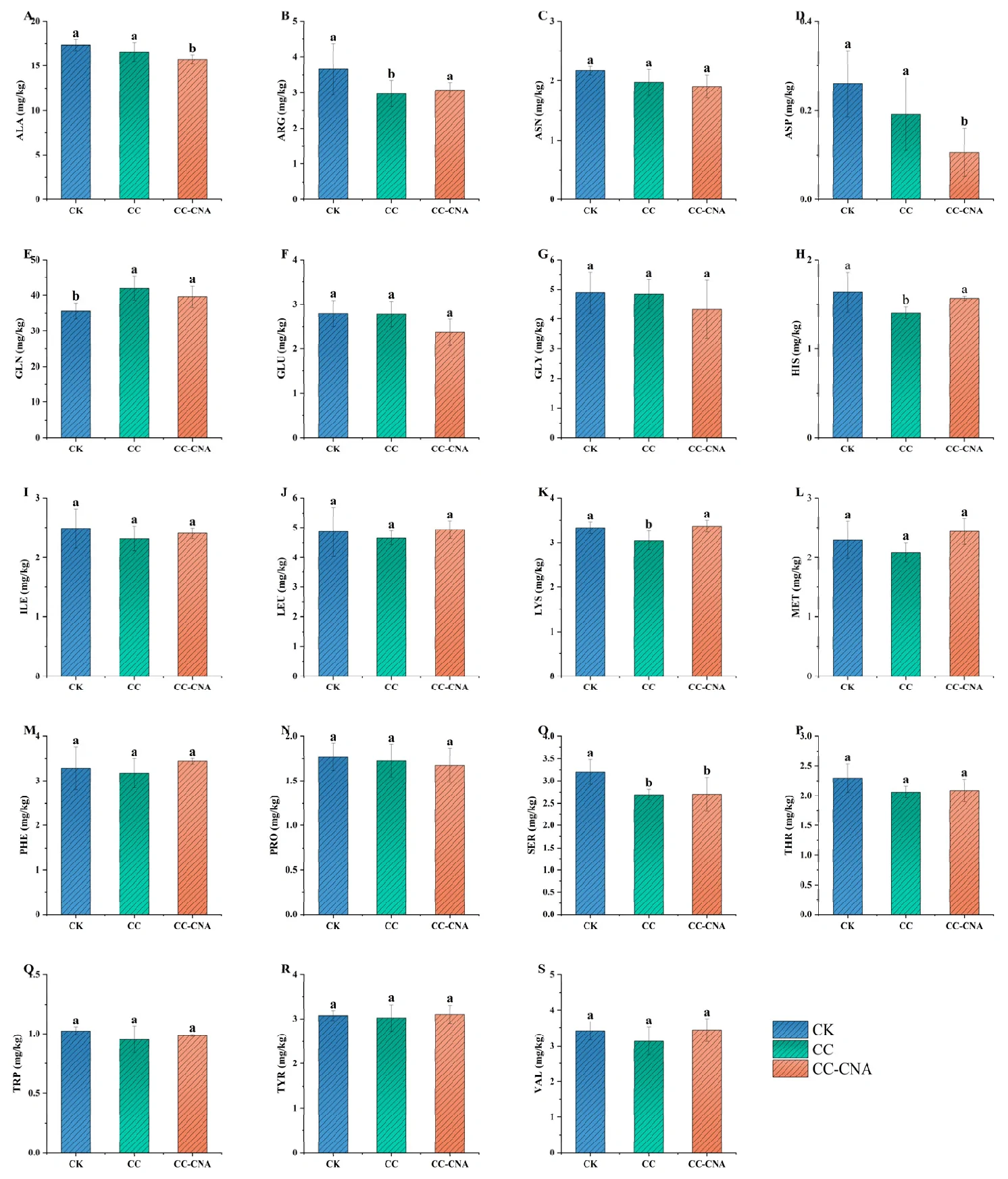
Distribution of free amino acid contents measured in beef among the experimental groups. Different letters identify values showing significant differences at *p* < 0.05. (**A**) ALA, Alanine; (**B**) ARG, Arginine; (**C**) ASN, Asparagine; (**D**) ASP, Aspartic acid; (**E**) GLN, Glutamine; (**F**) GLU, Glutamic acid; (**G**) GLY, Glycine; (**H**) HIS, Histidine; (**I**) ILE, Isoleucine; (**J**) LEU, Leucine; (**K**) LYS, Lysine; (**L**) MET, Methionine; (**M**) PHE, Phenylalanine; (**N**) PRO, Proline; (**O**) SER, Serine; (**P**) THR, Threonine; (**Q**) TRP, Tryptophan; (**R**) TYR, Tyrosine; (**S**) VAL, Valine. *n* = 8 per group.

Nineteen free amino acids were detected in beef samples from the three experimental groups, including essential alongside other nonessential amino acids. As shown in Figure 3, supplementation did not significantly affect asparagine, glycine, glutamic acid, threonine, proline, tyrosine, methionine, valine, isoleucine, leucine, phenylalanine, or tryptophan contents (*p* > 0.05). By contrast, glutamine was significantly higher in CC and CC-CNA than in CON (*p* < 0.05), while glutamate displayed a nonsignificant downward tendency. Serine occurred at a lower level in both supplemented groups relative to CON (*p* < 0.05). Aspartic acid and alanine showed significant decreases in CC-CNA relative to the other treatments, whereas arginine, histidine, and lysine were significantly reduced in CC (*p* < 0.05).

### 3.6. Meat Quality

Among the measured meat-quality variables, the L*, a*, and b* color coordinates, cooking loss, together with intramuscular fat content exhibited no statistically meaningful differences among groups (*p* > 0.05). The shear-force measurement, however, yielded significantly lower values for both CC and CC-CNA than for CON (*p* < 0.05). The lower shear force is consistent with a potential improvement in meat tenderness (Table 6).

## 4. Discussion

Changes in body weight are not necessarily observed following dietary RPC or NA supplementation. In the present experiment, IBW, FBW, and ADG showed no statistically significant differences across the three treatment groups examined. Li et al. [6] likewise reported no changes in IBW, FBW, or ADG after supplementing Ao-hu male sheep with 0.25% RPC for 75 days. Islam et al. [21] also observed no significant effect on ADG after 56 days of NA supplementation in preweaning dairy calves. The lack of a significant growth response in this study could be associated with the limited feeding duration, individual variability, or compensatory metabolic mechanisms. Choline is involved in lipid metabolism and methyl-group transfer, whereas nicotinic acid participates in redox reactions and energy metabolism. Given the absence of a significant growth response, larger studies with longer feeding periods and clearly defined supplementation schedules are needed to determine whether these nutrients affect growth performance.

The RPC might influence the composition of membrane lipids in muscle and intracellular lipid metabolism [22], but in this study, the effect of NA was more significant. The NEFA and MDA values of the CC-CNA group were significantly lower than those of the CC group, while TG value was significantly higher than that of the CC group. RPC insufficiency may lead to mitochondrial dysfunction and increased oxidative stress in livestock [6]. Although NA supplementation may restore intracellular energy levels and induce antioxidant synthesis [21,23], evidence for this in livestock is still lacking. Studies on dairy cattle show that RPC and NA may affect blood glucose and lipid metabolism, possibly having anti-inflammatory effects and stimulating mitochondrial biogenesis [6,13,24,25,26]. In this study, dietary supplementation with RPC and NA resulted in a significant increase in the serum levels of SOD and GSH-Px, in agreement with an antioxidant response. However, because markers of lipogenesis, steatosis, and stress were not measured in this study, the metabolic significance of the elevated TG levels remains unknown.

The CC treatment resulted in a different metabolite profile from that of CON, mainly characterised by L-tryptophan, L-proline and indole derivatives. Earlier work has indicated that choline may affect amino acid stores and neuroendocrine communication through the tryptophan-serotonin metabolic pathway and may also affect nitrogen utilization [27,28,29]. The CC-CNA treatment, in contrast, showed adenosine, uric acid and phytosphingosine as significant distinguishing metabolites. Pathways related to energy homeostasis, such as AMPK and lipolysis regulation, nucleotide metabolism, vascular activity and hormonal regulation [30,31,32] are also included. The supplementation did not reverse the overall trend of downregulation observed with choline alone. Adenosine, a unique CC-CNA metabolite, may reflect altered energy expenditure and intercellular signaling, consistent with trends in AMPK signaling and lipolysis regulation [33]. Choline appears to be involved primarily in amino acid metabolism and nitrogen transport, whereas the combined treatment is associated with a broader range of pathways, including lipid metabolism, nucleotide metabolism and signal transduction. The documented systematic metabolic responses provide a basis for exploring possible links between supplementation and meat-quality traits. A single fasting blood sample was used in this study, which may not fully reflect dynamic metabolic changes. Repeated sampling designs, as exemplified by İnanç and Arık [34] in fattening ruminants, would provide a more complete metabolic profile in future work.

Rumen microbial structure and function are strongly influenced by forage composition and digestibility [35]. Wei et al. [36] proposed that forage-induced remodeling of the rumen microbiome can alter meat protein and fatty-acid profiles and potentially influence meat-quality outcomes. In the present study, 23 bacterial phyla were identified, with Bacillota and Bacteroidota predominating as expected for the ruminant rumen microbiome [37,38]. Succiniclasticum was the dominant genus. According to Hu et al. [39], members of this genus ferment sugars, contribute to ruminal energy utilization, and become enriched in energy-intensive diets. Yang et al. [17] reported increased relative abundances of two Fibrobacter succinogenes taxonomic units in cattle receiving 1200 mg/kg NA. Fibrobacter succinogenes contributes substantially to ruminal cellulose digestion, producing formate, acetate, and succinate during cellulose degradation [40]. The present work evaluated how dietary NA and RPC supplementation affected the rumen microbiome of late-fattening beef cattle, but small sample size limits statistical power for omics analyses. These findings may help inform feed-additive strategies for modulating rumen microbiota, with potential implications for feed efficiency.

Amino acids contribute to meat taste and aroma both directly and as precursors of flavor compounds [41]. Free amino acids occur at relatively low concentrations in fresh meat but can influence taste perception and subsequent flavor formation [42]. Their sensory contribution varies with amino acid identity and concentration. Bitter taste is generally associated with histidine, tyrosine, methionine, valine, isoleucine, leucine, phenylalanine, and tryptophan, whereas serine, glycine, threonine, alanine, and proline are linked to sweet taste [43,44]. Aspartic acid and glutamic acid contribute to umami; arginine and lysine have sweet/bitter characteristics, while asparagine and glutamine are relatively neutral [45,46]. The treatment-related changes in free amino acid contents suggest that choline and nicotinic acid supplementation may alter amino acid metabolism in cattle. Nicotinic acid can be converted to nicotinamide in vivo and may increase NAD+ concentrations [47]. Through effects on energy metabolism and redox balance, nicotinic acid may modify enzymes involved in amino acid metabolism and thereby favor the conversion of glutamate to glutamine [48,49]. Choline metabolism generates betaine and phosphatidylcholine, which serve as a methyl-group donor and as a structural component of intestinal epithelial cell membranes, respectively, and may consequently influence nutrient absorption [50,51]. The observed changes therefore indicate an effect of supplementation on taste-related free amino acid profiles. However, amino acid composition alone provides only an indirect measure of sensory perception. Because no direct sensory evaluation, electronic tongue analysis, or taste activity value analysis was performed, inferred effects on bitterness and umami require cautious interpretation. Future studies should pair amino acid profiling with sensory evaluation, using electronic tongue technology or actual sensory panels to further confirm changes in taste.

Previous work has shown that dietary RPC can reduce shear force together with drip and cooking losses in post-slaughter muscle [6,7,8], whereas the present study detected a significant reduction only in shear force. Dietary NA has also been reported to promote fat deposition [9,11], but the increase in backfat thickness observed here did not reach statistical significance. Both CC and CC-CNA reduced shear force significantly. Because lower shear force generally corresponds to greater beef tenderness [52], this result supports a tenderness-related effect of supplementation. In contrast, L*, a*, b*, cooking loss, pH, and intramuscular fat content were unaffected by treatment. The meat-quality response should therefore be interpreted primarily in terms of tenderness rather than as a generalized improvement in meat quality. Because NA was provided only during the final 30 days, this design cannot separate the long-term effects of RPC from the short-term effects of NA. Future studies should provide both additives throughout the treatment period to fully evaluate their combined effects.

## 5. Conclusions

In conclusion, both CC and CC-CNA significantly decreased shear force, suggesting a potential improvement in beef tenderness. Exploratory serum metabolomics identified candidate treatment-associated metabolites and enriched pathways; however, these findings require validation. Supplementation also altered selected taste-related free amino acids, but direct sensory evaluation is required to establish whether these compositional shifts lead to flavor differences. Rumen-protected choline alone or combined with nicotinic acid may therefore influence meat tenderness and systemic metabolic responses in late-fattening beef cattle. Future work should identify appropriate supplementation doses and durations and clarify the underlying mechanisms in larger populations through integrated multi-omics analyses.

## Figures and Tables

**Table 1:** Basal-ration composition and nutrient concentrations (reported on an air-dry basis).

Ingredients	Content (%)	Nutrient Levels	Content (%)
Barley	16.00	DM	90.66
Corn	44.00	CP	10.95
Soybean meal	6.40	EE	2.43
Wheat bran	9.60	ADF	11.90
Straw	20.00	NDF	54.05
Premix	4.00	NE_mf_ (MJ/kg)	12.43
Total	100.00		

This premix supplied, for each kilogram, 93.98 g salt, 9000 IU Vitamin A, 200 IU Vitamin D3, 16 IU Vitamin E, 100 mg iron, 54 mg manganese, 54 mg zinc, 9 mg copper, 6 mg iodine, 0.2 mg selenium, 1 mg cobalt, 0.75 g calcium, and 0.15 g phosphorus. Abbreviations: DM denotes dry matter; CP denotes crude protein; EE, crude fat; ADF denotes acid detergent fiber; NDF denotes neutral detergent fiber. The NE_mf_ was the calculated value, and the remaining nutritional levels were the measured values.

**Table 2:** Influence of choline and nicotinic acid supplementation on growth outcomes in late-fattening cattle.

Items	Groups			SEM	*p*-Value
	CON	CC	CC-CNA		
IBW (kg)	748.38	770.17	758.99	8.94	0.64
FBW (kg)	802.80	825.00	818.20	7.32	0.48
ADG (g/d)	604.67	609.22	657.89	23.76	0.53

IBW denotes initial weight, FBW denotes final weight, and ADG denotes average daily gain. *n* = 8 per group.

**Table 3:** Influence of choline and nicotinic acid supplementation on serum biochemical indices of late-fattening cattle.

Items	Groups			SEM	*p*-Value
	CON	CC	CC-CNA		
NEFA (mmol/L)	0.69 ^a^	0.62 ^b^	0.50 ^c^	0.01	<0.01
TG (mmol/L)	0.24 ^b^	0.25 ^b^	0.27 ^a^	<0.01	<0.01
BUN (mmol/L)	18.29 ^a^	16.98 ^b^	16.68 ^b^	0.18	<0.01
ALT (U/L)	45.30 ^a^	39.40 ^c^	43.14 ^b^	0.50	<0.01
SOD (U/mL)	212.03 ^b^	216.31 ^a^	218.01 ^a^	0.86	0.01
GSH-Px (U/mL)	1548.01 ^b^	1588.43 ^a^	1612.12 ^a^	7.52	<0.01
MDA (nmol/mL)	7.71 ^a^	7.59 ^ab^	7.31 ^b^	0.06	0.03

Different letters identify values showing significant differences at *p* < 0.05. Abbreviations: NEFA denotes non-esterified fatty acids; TG, triglycerides; BUN, blood urea nitrogen; ALT denotes alanine aminotransferase; SOD denotes superoxide dismutase; GSH-Px denotes glutathione peroxidase; MDA denotes malondialdehyde. *n* = 8 per group.

**Table 4:** KEGG enrichment pathways of differential metabolites.

Comparison Group	Metabolic Pathway	Differential Metabolites	*p*-Value
CC vs. CON	Protein digestion and absorption	L-Tryptophan; L-Proline; Indole	<0.01
	Tryptophan metabolism	L-Tryptophan; Indole; Methylindole	<0.01
	Aminoacyl-tRNA biosynthesis	L-Tryptophan; L-Proline	<0.01
	Mineral absorption	L-Tryptophan; L-Proline	<0.01
	Phenylalanine, tyrosine, and tryptophan biosynthesis	L-Tryptophan; Indole	<0.01
	Central carbon metabolism in cancer	L-Tryptophan; L-Proline	<0.01
	Biosynthesis of amino acids	L-Tryptophan; L-Proline	<0.01
	Porphyrin metabolism	Bacterial Chlorophyllide A; 3-Hydroxyethyl Chlorophyll	<0.01
	Serotonergic synapse	L-Tryptophan	0.04
CC-CNA vs. CON	Purine metabolism	Uric Acid; Adenosine	<0.01
	Regulation of lipolysis in adipocytes	Adenosine	<0.01
	cGMP-PKG signaling pathway	Adenosine	<0.01
	Sphingolipid signaling pathway	Adenosine	<0.01
	Sphingolipid metabolism	Phytosphingosine	<0.01
	Vascular smooth muscle contraction	Adenosine	<0.01
	AMPK signaling pathway	Adenosine	0.01

**Table 5:** Effects of choline and nicotinic acid supplementation on rumen bacterial alpha diversity in late-fattening beef cattle.

Items	Groups			SEM	*p*-Value
	CON	CC	CC-CNA		
Sobs index	1363.80	1396.67	1370.71	43.46	0.96
Chao1	1692.80	1730.63	1716.23	49.27	0.96
ACE	1678.61	1722.55	1709.79	47.76	0.94
Shannon	5.24	4.92	4.88	0.11	0.40
Simpson	0.02	0.05	0.06	0.01	0.22
Good’s coverage (%)	99.09	99.10	99.10	0.04	0.99

*n* = 8 per group.

**Table 6:** Influence of choline and nicotinic acid supplementation on slaughter performance together with meat quality of late-fattening cattle.

Items	Groups			SEM	*p*-Value
	CON	CC	CC-CNA		
Carcass weight (kg)	440.76	445.96	450.73	11.16	0.94
Lightness L*	33.23	34.91	34.27	0.59	0.54
Redness a*	17.21	17.09	18.07	0.90	0.90
Yellowness b*	10.25	10.90	10.00	0.64	0.86
Shear force (N)	69.76 ^a^	47.86 ^b^	49.23 ^b^	4.22	0.03
Cooking loss rate (%)	15.68	16.08	16.14	1.03	0.98
Intramuscular fat (%)	30.36	37.15	35.89	11.42	0.47

Different letters identify values showing significant differences at *p* < 0.05. *n* = 8 per group.

## Data Availability

The rumen microbiome 16S rRNA gene V3–V4 amplicon-sequencing dataset generated in this study has been deposited in the NCBI Sequence Read Archive under BioProject accession PRJNA1533965 and SRA Study accession SRP739513. The BioSample accessions are SAMN63423032–SAMN63423054, and the SRA Run accessions are SRR40816318–SRR40816340. Metabolomics data has been uploaded to the MetaboLights database. The login link is "https://www.ebi.ac.uk/metabolights/reviewer395ba4b1-744c-4eb1-ac44-01611dea9fc8" and the password is "mtbls_94dc98e6e1e1e895ee339cb62466b37f4d914e21c6229521ea2e2bafa676fc49". The above content have been added in the text.
